# The uses of provincial administrative health databases for research on palliative care: Insights from British Columbia, Canada

**DOI:** 10.1186/1472-684X-4-2

**Published:** 2005-02-17

**Authors:** Diane E Allan, Kelli I Stajduhar, R Colin Reid

**Affiliations:** 1 Centre on Aging, University of Victoria, Sedgewick A104, PO Box 1700, STN CSC, Victoria, BC, V8W 2Y2, Canada; 2 Centre on Aging and School of Nursing, University of Victoria, Sedgewick A104, PO Box 1700, STN CSC, Victoria, BC, V8W 2Y2, Canada; 3 Centre for Population and Health Services Research, Okanagan University College, 3333 University Way, Kelowna, BC, V1V 1V7, Canada

## Abstract

**Background:**

Research indicating that people increasingly prefer to die at home suggests that palliative care is likely to play a more prominent role in the future of Canada's health care system. Unfortunately, at a time when research evidence should be informing policy and service delivery, little is known about health service utilization by Canadians at the end of life. One existing mechanism that can help address this gap is provincial administrative health data. The purpose of this study was to explore the potential of administrative health data to identify characteristics of palliative care users, patterns of formal service utilization and predictors of palliative care use.

**Methods:**

Bivariate and multivariate analyses were used to examine data from the Capital Health Region, British Columbia Linked Health Databases for the period 1992/93 to 1998/99. The databases examined include continuing care, physician claims, hospital separations, and vital statistics. As the name implies, these databases can be linked at the individual level using unique identifiers so that health services utilization can be tracked across sectors.

**Results:**

General patterns of service use among palliative care patients suggest that general practitioner and medical specialist visits have decreased over time and the utilization of hospital beds has increased. Utilization of community-based services (i.e. home support and home nursing care) shows an overall pattern of decline. However, when compared to non-palliative care patients, palliative care patients spent fewer nights in hospital, used fewer hours of home support, and had a greater number of home nursing care visits.

**Conclusions:**

Administrative health databases can provide valuable information for examining service utilization patterns over time. However, given that decisions surrounding the designation of palliative care include factors beyond the scope of administrative databases (such as quality of life, personal preferences, social support), these databases should only be seen as one source of information to inform service delivery and policy decision making.

## Background

Despite the fact that we all die, the philosophy and practice of palliative and end of life care is relatively new [1]. Certainly, in the broader context of the Canadian health care system, and within most western cultures, palliative and end of life care has played a secondary role due in part to the dominance of the biomedical model and its inherent focus on cure [2,3]. Care at the end of life is likely to play a more prominent role, however, in the future of Canada's health care system [4-6]. This expectation is based on the projected increase in numbers of older persons and the associated heightened risk of developing age-related chronic diseases such as cancer and cardiopulmonary disease. Conjoint with this expectation, the focus of care is shifting from institutional to community care. Thus, as governments seek to contain health care costs and caregivers search for improved quality of life for those nearing the ends of their lives, effective palliative and end of life care will become a more prominent research and policy issue.

Quality palliative and end of life care has emerged as a core value of Canada's health system [7]. Indeed, over the past few years, the Canadian government, policymakers and health care professionals have responded to increasing concerns about the quality of care for the dying. In 2001, the Canadian government appointed a Minister with Special Responsibility for palliative care and a national palliative care Secretariat was established within Health Canada [8]. A national action plan on palliative and end of life care was subsequently developed and several national subcommittees and working groups have formed to deal specifically with issues related to health care services at the end of life. One of these issues is the lack of research and data to inform health service delivery and policy decision-making in palliative and end of life care [7].

At a time when research evidence should be informing policy and health service delivery, little is known about health service utilization by Canadians at the end of life. Although countries such as Australia and the United Kingdom have established national palliative care (PC) surveillance data and extensive work has been done in the United States to identify key data elements pertaining to the end of life, Canada has been slower to develop in this area. Many Canadian PC programs have developed regional data systems [9], and a core data set for national surveillance is currently being developed [10]. National data standards and a surveillance system, however, are yet to be fully implemented in Canada. Therefore, researchers are examining existing sources of data to assist in informing health service and policy decisions. One existing mechanism that may assist in informing such decisions is provincial administrative health databases.

The primary purpose of this paper is to explore the usefulness of provincial administrative health databases as a source of information to inform health care decision making and policy development in palliative and end of life care. Data from the Capital Regional District in British Columbia, Canada are examined for the period 1992/93 to 1998/99. Specifically, our analyses identify characteristics of PC patients, patterns of formal service utilization over time, and predictors of PC service utilization. The implications of our findings for both research and policy, along with recommendations for future data collection initiatives are addressed.

### Palliative care: Patient characteristics and service utilization

Research on dying persons has grown in the past decade due, in part, to a rapidly aging population, policy concerns about increasing health expenditures, and a perception that the quality of care at the end of life is inadequate [11,12]. Several patterns have emerged describing patients who receive specialized PC. In Canada and the United Kingdom, for example, over 90 percent of palliative service users have a primary diagnosis of cancer [13]; 70–90 percent of palliative service users in Australia and more than 60 percent in the United States have likewise been diagnosed with cancer [13,14].

Levels of PC services utilization tend to be similar for both males and females [13,15,16,18] although there are exceptions. For example, Grande and associates (1998) reported that more males than females used PC services, while a later study by Grande and colleagues (2002) found more females used PC services. Generally, the relationship between age and PC service use is consistent across studies with services typically provided to adults in the younger older age range (i.e. 65–74 years) [13,15,17-21]. Results of studies investigating the relationship between socioeconomic status and PC service use have not been consistent with some studies reporting a positive relationship between income and referral to PC services [17,22,21,23] while others report no significant relationships [15,18,24].

Some research has examined the patterns of formal service utilization of PC patients. The more frequently cited measures of service utilization include scope of services used, along with length of stay (on a particular PC service) and location of death [4,13,24,25]. Yet, even these have received limited research attention. In Canada, population-based studies examining PC patients commonly use vital statistics and/or cancer registry data [4,22,25]. While providing important information, both data sources are limited in scope. Mortality data typically include underlying cause of death and date of death but little information on demographic characteristics or service utilization. Cancer registries contain demographic and service utilization variables, but are restricted to capturing only data on cancer patients and therefore exclude patients dying from other diseases [26].

### Administrative health databases

While administrative health databases have been used for research purposes for decades in Canada, it was the establishment of the Manitoba Centre for Health Policy and Evaluation and the subsequent development of their population health information system, POPULIS [27], which brought this data source into the spotlight. Similar developments in other Canadian provinces since that time now offer health researchers access to provincial administrative health data that can be linked across services. In British Columbia, the Centre for Health Services and Policy Research maintains and links provincial health data and is responsible for extracting and providing data to health researchers upon request from and approval by the Ministry of Health [28]. Like mortality and cancer registry data, however, there are limitations to administrative health data. These databases, for example, were originally constructed to serve a billing role; service providers submit claims in order to be reimbursed for services provided. The data contained in these datasets were thus not originally intended for research purposes. Therefore, while the databases are rich in information for select utilization, supply, and cost issues, their usefulness for addressing other factors that may influence the health of populations is limited. In the context of palliative and end of life care, it is this final point that bears further examination. Specifically, do these administrative databases identify persons receiving PC, and if so, what information is available to researchers interested in studying PC patients and their health service utilization? In addressing these questions, we are primarily concerned with providing examples of what can be done with these data rather than what the data are actually telling us about PC patients and their health care utilization.

## Methods

Data for this paper came from the British Columbia Linked Health Database. Housed at the University of British Columbia (BC), this database contains all provincial administrative health data collected by the BC Ministry of Health including physician claims, hospital separations, long-term care data, pharmacare data and vital statistics. Each of these components can be analyzed separately, as a stand-alone database, or linked through unique identification numbers and examined in combination or as a whole. The data were originally collected as part of a larger project examining the impact of regionalization in BC from 1990/91 to 1998/99 [29]. However, as the purpose of this particular paper fell outside the objectives of the larger study, a separate request to the BC Ministry of Health for data access was submitted and approved. Ethics approval from the academic institution was also received.

Given that PC patients could not be identified in the data prior to 1992/93, for the purposes of this paper, data from fiscal years 1992/93 to 1998/99 for all databases (with the exception of vital statistics where only 1998/99 figures were available) were included. Results are based on a 100 percent sample of the population aged 50 and over residing in the Capital Regional District, on Vancouver Island, British Columbia, Canada for each of the years. Both univariate and multivariate analyses were used to determine the usefulness of administrative databases for PC research in British Columbia.

The starting point for these analyses was the identification of all patients designated as being in need of PC (it should be noted that not all persons who may require PC are identified as needing it in the databases, nor are all people who use PC services included in the databases). This was made possible through the 'service type' variable found in the direct care services database, one of many databases that make up the continuing care component of the administrative databases. Next, using the unique identification numbers that are consistent across the different databases, it is possible to link these different datasets thereby allowing for the examination of individual service utilization across the various segments of the health care system (specifically: physician claims, hospital separations, and the home support and home nursing care components of continuing care).

The continuing care database is a suite of databases, however for the purpose of this paper only the direct care services and home support databases were accessed. The direct care services databases were used in this paper for 2 purposes. First, and most importantly, it allowed us to identify those designated palliative through an individual variable. It should be noted that those never entering the continuing care system but who receive palliative services in another sector (i.e., entered the hospital, received palliative services and died in hospital) could not be identified as such and thus are not designated palliative for these analyses. Second, the direct care services database also contains information on home nursing care. For this paper, the number of home nursing visits received by each individual in a given year was calculated. Home support utilization is tracked on a monthly basis. From these files it was possible to calculate the number of hours of home support received by each individual in a given year.

All claims made by physicians are tracked in the Medical Services Plan database. It is therefore possible to calculate the number of visits to a physician made by individuals over the course of a year. The hospital separations database is so named because a hospital patient is entered into the system only when they leave the hospital (this includes deaths). Since admission and separation dates are included as variables, it is possible to calculate the number of nights each individual spends in the hospital (no overnight stay is assigned a 0). Total nights in a given year for each individual can then be summed. The vital statistics data that was accessed included underlying cause of death. This variable is comprised of ICD-9 codes that can be classified into disease categories. This procedure allowed for the identification of cancer versus non-cancer deaths used in the multivariate analysis.

Each of these health databases includes a small number of demographic variables such as age and gender, and geographic identifiers such as health authority and census tract. Marital status is included in one of the continuing care databases. This lack of what are typically referred to as 'control variables' is one of the biggest limitations of administrative databases. To compensate, it is possible to link Census variables at an aggregate level to the administrative data. Of course this procedure only works for variables that lend themselves to averages. For example, it is possible to calculate the average household income for an area but it is not possible to calculate the average gender for an area. Using this premise, average household income was linked to the administrative data at the enumeration area level (the smallest geographic unit released by Statistics Canada and the BC Ministry of Health). In brief, the average household income for an enumeration area is assigned to an individual residing anywhere within the enumeration area. Thus, income is an aggregate measure while the remainder of the variables used in this paper are individual measures.

Finally, as mentioned earlier, each of these databases is linkable. The term linkable is used to describe the assignment of the same unique identification number to an individual regardless of database. In other words, Person A will be assigned the same number in the hospital database and the continuing care database. As such, linkable databases allow researchers to examine health service utilization and health status of the same individual across sectors. Without the ability to link, it would be impossible, for example, to examine underlying cause of death (vital statistics database) in relation to the designation of palliative care (continuing care database).

As described in the preceding paragraphs, 5 health service utilization measures were calculated: number of general practitioner visits per year; number of medical specialist visits per year; number of nights spent in hospital per year; number of hours of home support received per month; and number of home nursing care visits received per month. Next, using these variables, three different analyses were conducted:

1. Descriptive statistics (distribution, mean, median) were used to examine PC patient characteristics and health service utilization by gender. All those identified as being in need of PC for each fiscal year were examined by age, gender, income, and the 5 health service utilization measures. The sample size of PC patients ranged from a low of 74 in 1992/93 to a high of 568 in 1997/98.

2. Because service use is likely to differ by diagnosis, we compared patients designated as being in need of PC with patients who were not designated as in need of PC in order to control for diagnosis. Previous research indicates that the majority of persons receiving specialized palliative and end of life care have a cancer diagnosis [13]. While it is acknowledged that different types of cancer place different demands on the health care system, for the purposes of this study, all cancers were examined in combination. Using the underlying cause of death code from vital statistics available for 1998/99, all those who died of cancer in 1998/99 (n = 2,734) were identified. Next, comparisons involving age, gender, income, and the 5 health service utilization measures were then made between cancer patients designated palliative (n = 119) and cancer patients not designated palliative (n = 2,615).

3. To examine the influence of a PC designation on health service utilization, five multiple linear regression models were estimated using the 1998/99 data from the palliative and non-palliative sample (n = 2,734) described above. The following five dependent variables were regressed on age, gender, average household income, and designation of palliative/non-palliative: (1) number of general practitioner visits; (2) number of medical specialist visits; (3) number of nights spent in the hospital; (4) number of hours of home support; and (5) number of home nursing care visits. Prior to conducting the multivariate analyses, assumptions of normality, linearity, and collinearity were tested and adjustments were made where necessary.

## Results

### 1. Palliative care patient and health service utilization characteristics

The linked administrative database allowed for a description of patient characteristics including age, gender, and income. Table 1 presents characteristics of PC patients by gender from 1992/93 to 1998/99. Results indicate that almost 40 percent of male and female PC patients are between the ages of 70 and 79 years and this trend is consistent across years for males, and varies from 34% to 45% in females. Median values for annual household income range from $46,757 to $53,377 for males and $41,923 to $48,753 for females over the study period. With the exception of 1992/93 and 1997/98 where the median income for males is substantially higher than that of females, the income differences between males and females are slight.

**Table 1:** Palliative Care Patient Characteristics by Gender: 1992/93–1995/96

	1992/93		1993/94		1994/95		1995/96	
	
	M (n = 42)	F (n = 32)	M (n = 212)	F (n = 178)	M (n = 225)	F (n = 194)	M (n = 207)	F (n = 216)
Age (%)								
50–59	9.6	3.1	13.2	9.5	6.7	13.9	10.1	6.5
60–69	21.4	18.8	21.7	32.0	25.7	25.8	23.7	21.7
70–79	47.6	46.9	39.7	34.3	42.7	39.1	38.7	39.4
80+	21.4	31.2	25.4	24.2	24.9	21.2	27.5	32.4
								
Median income	53377	41923	47682	46997	47272	48753	47238	47272
Palliative Care Patient Characteristics by Gender: 1996/97–1998/99								
		
	1996/97		1997/98		1998/99			
			
	M (n = 209)	F (n = 189)	M (n = 245)	M (n = 209)	F (n = 189)	F (n = 194)		
		
Age (%)								
50–59	8.6	9.4	6.6	8.6	9.4	13.9		
60–69	27.2	14.9	14.6	27.2	14.9	25.8		
70–79	39.2	45.5	40.4	39.2	45.5	39.1		
80+	25.0	30.2	38.4	25.0	30.2	21.2		
								
Median income	46757	45896	49984	46757	45896	48753		

The means for the use of the five health services are presented in Table 2. T-test results suggest that there are few health service utilization differences between male and female PC patients. General patterns suggest that female PC patients spend more nights in hospital and receive a greater number of monthly home support hours and home nursing care visits than do their male counterparts, however, few of these gender differences reach statistical significance. One exception is number of home support hours where the results suggest that in three of the seven years examined, females receive a greater number of home support hours than males.

**Table 2:** Mean Palliative Care Patient Service Utilization by Gender: 1992/93 – 1998/99

	Annual GP visits		Annual specialist visits		Nights spent in hospital annually		Monthly hours of home support		Monthly home nursing care visits	
	M	F	M	F	M	F	M	F	M	F
1992/93	19.2	21.3	14.3	19.4	99.9	106.7	15.3	26.0	23.0	29.8
1993/94	15.0	15.7	13.8	13.3	102.9	185.5	21.9**	31.0	25.7**	26.7
1994/95	15.1	13.9	12.9	13.6	136.6	172.3	24.6	30.9	19.7	22.0
1995/96	15.3	14.9	12.7	12.4	139.1	176.0	24.9	26.9	21.5	22.5
1996/97	12.8	13.9	11.9*	14.6	138.1	211.4	26.2*	30.5	19.5	24.0
1997/98	6.8	7.3	6.8	6.8	205.9	313.4	31.8**	28.2	23.1	25.3
1998/99	3.0	3.0	1.3	1.3	117.9	150.1	18.9	27.4	17.3	21.7

*p < .05; **p < .01 (t-test results comparing males and females)

Health service utilization trends observed across the years show that the number of annual general practitioner and specialist visits is fairly steady until 1997/98 when there is a dramatic drop. In contrast, the number of nights spent in hospital steadily increases over the same period until 1998/99 when a sharp reduction in hospital bed utilization is observed. Patterns of home support and home nursing care utilization over time are more variable. Home support hours per month exhibit a pattern similar to number of hospital nights, although the increases and decreases are not as large. Number of home nursing care visits has fluctuated over the years and shows a general pattern of decline from 1992/93 to 1998/99.

### 2. Palliative care vs. non-palliative care cancer patients and health service utilization characteristics

Results comparing cancer patients designated as being in need of PC and cancer patients who were not designated as in need of PC are presented in Table 3. Findings suggest that there are no differences between palliative and non-palliative cancer patients in terms of gender, average household income, and number of general practitioner and medical specialist visits. Differences are observed between the two groups for age, number of nights spent in hospital, number of hours of home support, and number of home nursing care visits. Specifically, PC cancer patients spend fewer nights in the hospital (p < .001), use fewer hours of home support (p < .05), and have a greater number of home nursing care visits (p < .001) than do non-PC cancer patients.

**Table 3:** Cancer Patient Characteristics and Service Utilization by Designation of Palliative: 1998/99

	Palliative	Non-palliative
Age group (%)***		
50–59	7.7	8.5
60–69	26.6	17.0
70–79	35.3	37.8
80+	30.4	36.7
		
Gender (%)		
Male	47.6	47.4
Female	52.4	52.6
Income ()	51,718	49,804
GP visits ()	3.23	3.21
Specialist visits ()	1.21	1.34
Nights in hospital ()***	20.97	24.19
Home support hours ()*	105.48	172.60
Home nursing care visits ()***	21.66	6.84

* p < .05; ** p < .01; *** p < .001 (X^2 ^and t-test results comparing palliative and non-palliative)

### 3. Predictors of health service utilization

Regression results for the five health service utilization regression models are presented in Table 4. Results suggest that age, gender, income, and designation of PC are not strongly predictive of health service utilization. There is a significant relationship between age and number of nights spent in hospital (p < .001), suggesting that the likelihood of spending a greater amount of time in the hospital increases with age. Being female (p < .01) and being older (p < .01) increases the amount of home support hours received, while being younger (p < .05) and being designated as being in need of PC (p < .001) increases the number of home nursing care visits received.

**Table 4:** Regression estimates and standard errors for five regressions

	GP visits		Specialist Visits		Nights in hospital		Home support hours		Home nursing care visits	
	
	B	SE	B	SE	B	SE	B	SE	B	SE
Age	.00	.00	-.00	.00	.14***	.01	.14**	.02	-.11*	.01
Gender	-.02	.01	-.01	.01	.05	.04	.12**	.06	.03	.05
Income	.01	.00	.00	.00	.00	.00	.04	.00	0.04	.00
Palliative	.02	.03	-.02	.02	-.05	.07	-.05	.10	.29***	.05
Adj. R^2^	-.001		-.001		.02		.03		.10	

* p < .05; **p < .01; ***p < .001

## Discussion

The primary purpose of this study was to explore the usefulness of provincial administrative health databases as a source of information to inform health care decision making and policy development in palliative and end of life care. Administrative databases represent an existing source of longitudinal information on health system users. The need to track individuals' health service utilization over time has long been recognized by researchers, and this is a key advantage of such databases. At the same time, however, the breadth of information collected is limited. For example, no direct measurements of quality of life, or for that matter, quality of death are compiled. The researcher who makes use of the databases must therefore work within these confines. The findings from the present study provide insights into the precise manner in which these databases can be of value to health services researchers, within these limits. More specifically, the analyses conducted for this paper exhibit that a sample of palliative patients can be identified in one of the many provincial administrative health databases. Since individuals can be linked across sectors, it is possible to examine a number of health status and utilization indicators of these individuals. Finally, linking the health databases with the vital statistics variables gives the researcher insight into the underlying cause of death.

The limitations of administrative databases arise directly from their intended purpose. They were created to serve a billing role, and although they are of value as a research tool, their limitations should be noted. First, socioeconomic variables are limited to age, gender and income. The importance of these variables is unquestioned; however, a wider range of such variables is normally a requirement for social science based health services research. Second, the precise date within a year that patients are designated palliative is not available. Since health service resource utilization has been shown to be much greater during the final few months and weeks of life, information on date designated palliative would be informative. Third, the data that would allow for an examination of either quality of care provided or quality of dying – both of which are central to any study seeking to improve service delivery and associated outcomes – is not collected. Finally, given the complexities of data collection within the health care system, these databases are unlikely to capture all individuals in need of PC. For example, given the fluctuation in the number of palliative patients across years, with special emphasis on the precipitous decline observed between 1997/98 and 1998/99, it appears that the definition and coding of the variable designating an individual palliative may be subject to change over time. If these data are to be used for research purposes, this indicates the need for careful monitoring for continuity of both definition and coding procedures.

While the primary objective of this paper was not to focus on the actual trends and findings, the results hold potential for future studies. For example, the observed decrease in both GP and specialist visits between 1992/93 and 1998/99 for males and females designated as being in need of PC may be indicative of changing trends. First, persons designated palliative are increasingly signaling their desire to die at home. Second, perhaps GPs are increasingly visiting dying patients at home, with the result that these visits are not captured in the health databases since physicians cannot bill for such visits. As GPs become more aware of the need for effective PC, and as they acquire the requisite skills to do so, the territory once thought to be the exclusive realm of the specialist may be experiencing erosion. In the larger picture, this may be indicative of a shift away from a curative medical model approach to end of life care, to a more appropriate social model.

At the same time that GP and specialist visits among persons designated as being in need of PC have declined, mean number of nights spent in hospital annually have shown a general increase. Ostensibly, the increase in hospital nights until the 1997/98 period is a puzzling trend: especially given findings on the general population that show a decrease in nights spent in hospital throughout the 1990's [30]. A potential explanation is that although the rhetoric surrounding the issue trumpets the need to orchestrate a "closer to home" health care delivery system for palliative persons, the reality is that the resources have not yet been committed. Consistent with this explanation, the sharp decline in hospital visits by palliative persons observed for the 1998/99 period may reflect an increased flow of resources towards effective community care. However, prior to making any conclusions about a shift in direction of resources and subsequent utilization patterns, it is necessary to examine the figures beyond 1998/99. In other words, does the decline in hospital nights continue or was this year simply an anomaly?

Although it is not monotonic, the general decline in monthly home nursing visits between 1992/93 and 1998/99 is also somewhat puzzling on the surface, given the calls to enhance home nursing services for the dying. A comparison of home nursing visits between palliative and non-palliative cancer patients, however, reveals that those designated palliative receive considerably more home nursing visits. This latter observation is consistent with the increased care requirements at the end of life. In trying to decipher the differences between these two groups it would be important to examine the time of the visits in relation to death. For instance, do more visits occur in the last months or weeks leading up to death?

In the multivariate analyses, it is of particular interest that being designated palliative is related only to number of home nursing care visits. In other words, being designated palliative has no bearing on general practitioner visits, medical specialist visits, number of hospital nights and number of hours of home support. This, of course, is contrary to bivariate findings presented in Table 3 that reveal significant differences between palliative and non-palliative patients in number of nights spent in hospital and number of hours of home support received. Thus, it appears that when age, gender and income are controlled for, the initial differences found between palliative and non-palliative individuals disappear.

## Conclusions

These data provide another piece of the overall picture of service utilization at the end of life. Research into palliative and end of life care is in its infancy at a time when effective and immediate information based on sound scientific research is urgently required. To the extent that administrative databases can bridge this gap, they are of immediate use. Their primary usefulness, however, is probably as a means of identifying and sketching a larger picture, and from that picture, facilitating the generation of key questions that may serve as platforms for launching more in-depth studies. For example, the decline over time in GP and specialist visits, combined with the general decline in home nursing visits for palliative persons raises the question of whether resources have met rhetoric, and if not, why not? Given that health care and policy decisions must include information on factors beyond the scope of administrative databases (such as quality of life, personal preferences, social support), these databases should only be seen as one piece of information to inform service delivery and policy decision making for patients and families at the end of life.

## Competing interests

The author(s) declare that they have no competing interests.

## Authors' contributions

All authors participated in the conceptualization and design of the study. DEA drafted the initial background, methods and results sections. KIS and DEA conducted all the analyses. KIS and RCR drafted the discussion section and provided additional edits to all sections. All authors read and approved the final manuscript.

## Pre-publication history

The pre-publication history for this paper can be accessed here:


